# Phage-Derived Peptidoglycan Degrading Enzymes: Challenges and Future Prospects for In Vivo Therapy

**DOI:** 10.3390/v10060292

**Published:** 2018-05-29

**Authors:** Hugo Oliveira, Carlos São-José, Joana Azeredo

**Affiliations:** 1 CEB—Centre of Biological Engineering, LIBRO—Laboratório de Investigação em Biofilmes Rosário Oliveira, University of Minho, 4710-057 Braga, Portugal; hugooliveira@deb.uminho.pt; 2 Research Institute for Medicines (iMed.ULisboa), Faculty of Pharmacy, Universidade de Lisboa, Av. Prof. Gama Pinto, 1649-003 Lisboa, Portugal; csaojose@ff.ul.pt

**Keywords:** bacteriophage-derived enzybiotics, virion-associated lysin, endolysin, in vitro, in vivo

## Abstract

Peptidoglycan degrading enzymes are of increasing interest as antibacterial agents, especially against multi-drug resistant pathogens. Herein we present a review about the biological features of virion-associated lysins and endolysins, phage-derived enzymes that have naturally evolved to compromise the bacterial peptidoglycan from without and from within, respectively. These natural features may determine the adaptability of the enzymes to kill bacteria in different environments. Endolysins are by far the most studied group of peptidoglycan-degrading enzymes, with several studies showing that they can exhibit potent antibacterial activity under specific conditions. However, the lytic activity of most endolysins seems to be significantly reduced when tested against actively growing bacteria, something that may be related to fact that these enzymes are naturally designed to degrade the peptidoglycan from within dead cells. This may negatively impact the efficacy of the endolysin in treating some infections in vivo. Here, we present a critical view of the methods commonly used to evaluate in vitro and in vivo the antibacterial performance of PG-degrading enzymes, focusing on the major hurdles concerning in vitro-to-in vivo translation.

## 1. The Antibiotic Resistance Crisis

Bacterial infections have always impacted human health and are among the leading cause of death [1,2]. This problem is recently becoming more critical, as we witness an unprecedented rise of multi-drug resistant pathogens that are considered by the Infectious Diseases Society of America and the European Society of Clinical Microbiology and Infectious Diseases one of the greatest threats to human health of the new millennium [3,4,5].

ESKAPE pathogens (*Enterococcus faecium*, *Staphylococcus aureus*, *Klebsiella pneumoniae*, *Acinetobacter baumannii*, *Pseudomonas aeruginosa* and other *Enterobacteriaceae* species) are the leading group among these so-called superbugs, that can rapidly gain resistances to several classes of antibiotics and are able to cause a variety of nosocomial infections (such as bacteremia, wound and skin infections) [6,7]. The reduced susceptibility towards antibiotics makes the treatment of ESKAPE infections extremely difficult and scientists are now trying to widen the repertoire of alternative antibacterial agents. Phage-encoded enzymes that have the capacity to degrade the major component of the bacterial cell wall (CW), the peptidoglycan (PG), or murein, represent a promising alternative class of antibacterial agents with increasing prominence [8,9]. Almost two decades after the first experimental validation of their antibacterial potential [10], we present the biological, molecular and antibacterial properties of the different phage-derived PG-degrading enzymes and highlight some advantages, constraints and future considerations useful when aiming at using the antibacterial potential of these enzymes (“enzybiotics”) against infectious diseases in vitro and in vivo.

## 2. Peptidoglycan-Degrading Enzymes—An Emerging Class of Antibacterial Agents

### 2.1. Biology and Diversity

Tailed phages are ubiquitous viruses that specifically infect bacteria in order to replicate. At the end of the reproductive cycle these viruses lyse the host cells to release the descendant virus particles to the extracellular space. Therefore, during the lytic cycle a phage needs to breach the bacterial CW twice, first to deliver its DNA (and sometimes a few proteins) to the host cell cytoplasm and at the end to liberate the virion progeny. Phage PG-degrading enzymes are employed in both steps. Those acting during viral DNA entry are carried in the virus particle and are often called virion-associated lysins (VALs) or virion-associated peptidoglycan hydrolases [11]. At the beginning of phage infection, VALs attack the CW of host cells from outside and are thought to promote a local degradation of the murein to facilitate penetration of the phage tail tube and injection of the viral DNA [12]. Nevertheless, the fact that some VALs like those from *S. aureus* phage phi11 and *E. coli* phage T7 showed to be dispensable for phage infection in the lab suggests that they may confer an advantage only under certain physiological conditions [13,14].

The enzymes responsible for host cell lysis are synthesized in the cytoplasm of infected bacteria and are designated endolysins. At the end of phage infection, most endolysins require the action of another phage-encoded protein—the holin—to be able to cross the cytoplasmic membrane and gain access to the CW. Holins are usually small hydrophobic proteins that oligomerize in the bacterial cytoplasmic membrane and, at the appropriate time, they form lethal holes that are large enough to allow endolysin escape to the CW. Once in this cell compartment, endolysins quickly degrade the PG polymers of the killed host bacteria, leading to abrupt osmotic cell lysis and subsequent release of progeny phages [15,16].

VALs and endolysins have evolved multiple enzymatic activities to specifically degrade the PG. The PG polymer is composed of alternating *N*-acetylmuramic acid (MurNAc) and *N*-acetylglucosamine (GlcNAc) residues linked by β-1,4 glycosidic bonds. Adjacent glycan strands are cross-linked by short interconnected stem peptides, each of them linked to MurNAc residues through an amide bond involving the first amino acid of the peptide stem (usually l-alanine [17]). Depending on the bonds that VALs or endolysins cleave, their activity can be categorized into different classes: lysozyme, transglycosylase, glucosaminidase, amidase or endopeptidase [18]. The first three classes cleave the glycosidic bonds in the glycan chains, amidases hydrolyze the amide bond connecting MurNAc to the peptide stems and endopeptidases cleave within the peptide moiety.

Endolysins can have a globular or a modular design [18,19]. In the first case, the lytic enzymes essentially correspond to the single enzymatic catalytic domain (ECD) responsible for cleaving a specific PG bond. In the most common modular structure, one or two N-terminal ECDs are connected by a flexible linker to one or more cell binding domains (CBD), which are responsible for recognizing specific epitopes on the CW. This modular configuration is most commonly found in endolysins of phages infecting Gram-positive bacteria and mycobacteria [8,18,20]. Nevertheless, few exceptions with modular structure have been reported in Gram-negative systems but, interestingly, with inverted orientation of the functional domains [21,22]. The diversity of endolysins is notorious with 24 and 13 different types of ECDs and CBDs, respectively, and 89 unique architectural organizations identified [18]. The most accepted hypothesis for the presence of CBD in most endolysins of phages infecting Gram-positive hosts, is that by being bound to the CW debris after cell lysis, the enzymes will not diffuse and destroy nearby potential hosts, therefore enabling progeny phages to initiate new infection rounds [23]. Since Gram-negative bacteria have an outer membrane (OM) protecting the internal PG, the endolysins released at the end of the phage lytic cycle should not compromise non-infected cells.

VALs are structurally much more diverse and usually larger (37–252 kDa) than cognate endolysins (15–40 kDa). They may confer structural and functional roles to phage virus particles in addition to murein cleavage and some are found as oligomers in the virion structure. VALs from Gram-positive systems frequently display two distinct ECDs to degrade the PG, likely as a response to the thick PG layer found on their hosts. There is one particular ECD, the endopeptidase ECD of the M23 family, which is frequently present in VALs and rarely found in endolysins. Interestingly, this domain is responsible for the PG-degrading activity of the bacteriolysins lysostaphin and enterolysin A [24,25], which also act from without and on actively growing cells. VALs however, usually lack CBDs [26]. The absence of CBDs in VALs could be explained by the fact that they are often subdomains of larger proteins or belong to virion structures that promote proximity of the VAL to the CW [26]. In fact, VALs often form part of tail fibers, tape measure proteins and baseplates (e.g., lactococcal phage TP901-1, *S. aureus* phage phiIPLA35 and *E. coli* phage T4, respectively) [27] but can also be internal capsid proteins (e.g., in *E. coli* phage T7 and *P. aeruginosa* phiKMV) [28].

### 2.2. Enzybiotics Lytic Activity and Efficacy

VALs lytic activity is responsible for the “lysis from without” phenomenon, first described in 1940, due to multiple phages puncturing [29]. In Gram-positive hosts, VALs from lactococcal phages Tuc2009 and TP901-1 and *Bacillus subtilis* phages SP-β and phi29 were tested and proved to degrade the bacterial CW [30,31]. Interestingly, the VALs of all these phages carry at least one ECD of the M23 family (peptidase). In the lactococcal phages this domain was shown to facilitate CW penetration and phage genome delivery, particularly to stationary phase cells that carry an extensively cross-linked CW [30]. VALs of *S. aureus*-infecting phages phiIPLA88 and P68 were shown to reduce ∼99% the cell counts of *S. aureus* clinical isolates after 20 min exposure, including methicillin-resistant strains [32,33]. Studies have also demonstrated the bifunctional lytic activity of phiMR11 VAL, bearing cysteine, histidine-dependent amidohydrolase/peptidase and lysozyme activities, by purifying each domain as recombinant proteins [34]. In Gram-negative pathogens, VALs activity has also been confirmed mostly on *Pseudomonas* hosts [35,36,37]. Using bacterial cells with permeabilized OM by chloroform treatment [21], the muralytic activity of these VALs showed to be exclusively against Gram-negative pathogens and in most cases independently of the phage host (e.g., *E. coli*, *P. fluorescens*, *P. putida)*. This is explained by the conserved composition and structure of the PG (chemotype A1γ) among Gram-negative bacteria, in contrast with the diverse chemotypes found in Gram-positives [38]. Some VALs are also highly thermostable. For instance, the *P. aeruginosa* phiKMV VAL could retain >20% of its activity after 2 h at 100 °C and even after autoclaving. *S. aureus* phage HydH5 VAL can resist 100 °C for 5 min [32]. This is probably related with their structural nature, evolved to endure harsh external conditions in order to maintain the phage infectivity. Additionally, VALs have been shown to synergize with endolysins and antibiotics [39,40]. Protein engineering to improve VALs performance has also been successful, as described for few *S. aureus* and *Enterococcus faecalis* phage enzymes [39,41,42,43]. Taken together, VALs have several appealing characteristics for biotechnological applications, particularly for those where thermal processing is required.

Endolysins have emerged over the last years as an exciting new antimicrobial and are far more studied than VALs. The first report of an activity of a recombinant endolysin dates back to 1959 [44]. Since then, nanogram quantities of certain endolysins have showed to be efficient in eliminating bacterial suspensions in seconds [10] and numerous studies have proved their antibacterial potential in vitro, for example against methicillin-resistant and multidrug-resistant *S. aureus* (endolysins LysK and MV-L, respectively) [45,46], and towards vancomycin-resistant *E. faecalis* and *E. faecium* (endolysin PlyV12) [47]. Endolysins have also been used in cocktails or in combination with antibiotics to eliminate pathogenic bacteria. Antibiotic-resistant *S. pneumoniae* strains have been targeted using Cpl-1 and Pal endolysins, which share the same target specificity but have different catalytic activities [48]. Synergistic effects have also been demonstrated with the Cpl-1 endolysin and penicillin or gentamicin antibiotics against *S. pneumoniae* strains with different levels of susceptibilities to penicillin [49]. Moreover, the need to develop endolysins with desired or enhanced properties for several biotechnological applications has led to the creation of tailor-made enzymes by adding, truncating or swapping functional domains (engineering strategies and major outcomes are reviewed in [50,51]). Endolysin engineering was for the most part pioneered by studies that highlighted the modular character of the lytic enzymes of *S. pneumoniae* and its phages. It was shown that ECDs and CBDs from different enzymes could be exchanged to generate chimeras that retained the native cleavage and binding properties of the individual modules [52]. In the case of staphylococcal phage endolysins, similar modifications were made to overcome solubility problems [53,54]. The CBD truncations of the *S. aureus* phage LysK and *Streptococcus agalactiae* PlyGBS endolysins resulted in 2- and ~25-fold increase in muralytic activity, respectively [55,56]. Elimination of endolysin CBD may additionally lead to the expansion of the enzymes’ spectrum of activity [57,58,59]. In an inverse approach, the addition of an extra CBD to the *Listeria* phage endolysin Ply500 increased CW binding affinity by approximately 50-fold, which translated in enhanced lytic activity in high salt conditions [60].

### 2.3. Recent Developments for Endolysins Targeting Gram-Negative Bacteria

While phage-derived PG-degrading enzymes have been shown to efficiently work exogenously as recombinant proteins to kill Gram-positive bacteria pathogens (for extensive review see [61]), their application against Gram-negative pathogens is more limited. As mentioned above, the presence of an OM acts as a protective barrier that prevents the enzymes added exogenously reaching the PG, making their application more challenging. In view of the conserved Gram-negative bacterial PG structure, the application of PG-degrading enzymes on these bacteria could be of great importance in the microbial control. Only recently, several studies have addressed this problem by (i) exploring the “natural” ability of some endolysins to kill Gram-negative bacteria, particular those possessing positively charged segments that are able to disorganize or even disrupt the OM (e.g., T4 and LysAB2 endolysins) [62,63], (ii) combining endolysins (e.g., EL188 and Lys68 endolysins) with OM permeabilizing agents such as EDTA and organic acids that displace divalent cations and destabilize the membranes [64,65,66], or (iii) genetically engineering endolysins like the so called Artilysins [67], which carry lipopolysaccharide-destabilizing peptides with a polycationic, hydrophobic or amphipathic nature (e.g., SMAP-29 and polycationic nonapeptide), to allow passage of the recombinant proteins through the OM [67,68]. These strategies were proven successful in vitro against a number of different pathogens, like *Salmonella*, *E. coli*, *A. baumannii* and *P. aeruginosa.* Artilysins (e.g., LoGT-008) have also been successfully applied in human keratinocytes and *Caenorhabditis elegans* models to control infections [67].

## 3. From Discovery to Therapeutic Application

**Figure 1:**
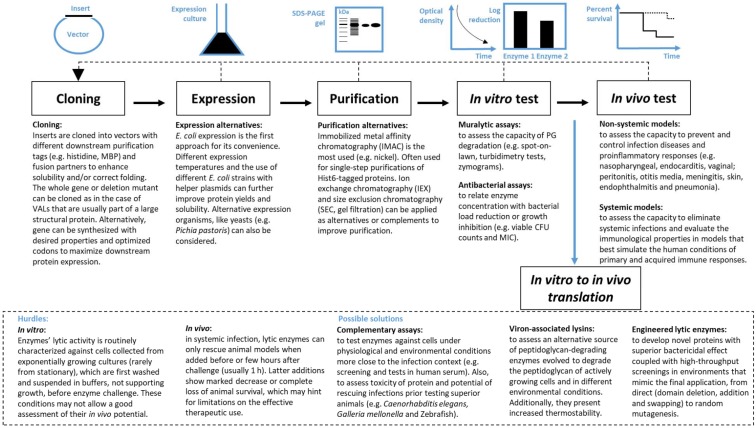
A step by step process for exploration and examination of the enzybiotic potential of phage-derived peptidoglycan-degrading enzymes. Five independent steps are identified in the flowchart, being the most common strategies used. An additional and intermedium step between in vitro and in vivo tests are discussed.

There is a great diversity of PG-degrading enzymes that can be harnessed to develop antibacterial agents. Figure 1 presents a flowchart with the typical steps underlying the exploration of phage lytic proteins. The process usually starts with in silico identification of genes encoding PG-degrading proteins in phage genome sequences. PG-degrading enzymes are well conserved and therefore easy to find bioinformatically using BLAST-based tools [69]. The next steps involve enzymes cloning, expression and purification of candidates, before evaluation of their in vitro and in vivo performance using several different possible methods as follows.

### 3.1. In Vitro Performance Evaluation

The in vitro assessment of PG-degrading activity can be performed directly or indirectly using different techniques. The spot-on-lawn method, the turbidimetry assay and zymogram analysis are most commonly used to directly evaluate lytic activity. Assessment of the remaining number of Colony Forming Units (CFUs), following enzyme testing and determination of minimum inhibitory concentrations (MIC) are often used to assess the impact of the enzybiotics on cell viability. These methods that distinctively evaluate bacteriolytic, bactericidal and bacteriostatic activities of enzybiotics have their own limitations, with some yielding inconsistent results when applied to certain lytic enzymes. For example, for reasons that are still unknown, some enzymes with high bacteriolytic activity seem not amenable to standard MIC determinations [8,70]. The selected protocol(s) will depend on the aim, state of enzyme characterization and easiness of implementation.

Due to its simplicity, the spot-on-lawn method is probably the first method performed to validate the putative muralytic function of a novel enzyme. There are two variants of this assay: (1) a diluted bacterial inoculum is spread on agar plates using overlay techniques and afterwards defined amounts of the enzybiotic in a small volume (usually 10 µL) are spotted on the lawn. Observation of clear halos, usually after overnight incubation, is indicative of growth inhibition as result of lytic activity [43]. Nevertheless, with Gram-positive bacteria this method can give false results if density of bacterial lawns is not controlled. Heavy growth of these bacterial cells can make them relatively resistant to the enzyme killing effect; (2) a concentrated bacterial inoculum is incorporated in an agarized physiologic buffer to prepare a ready-to-use, dense bacterial lawn. After spotting the enzybiotic, the bacteriolytic activity is revealed by the appearance of lysis zones after a few minutes or hours of incubation. This assay is useful to quickly determine the lytic spectra of the enzymes. In the case of Gram-negative species, an additional step involving treatment with chloroform vapors is needed to permeabilize the OM [65].

The turbidimetry assay is probably the most used method. Enzybiotics are incubated with suspensions of live, dead cells or PG extractions as substrate. In most cases live bacteria are used, which are grown to exponential phase and usually adjusted to an optical density of 1.0 prior to incubation with defined amounts of enzyme. Of note, in the vast majority of the cases the cell suspensions are prepared in buffered solutions, which are conditions not supporting bacterial growth and poorly mimicking in vivo infection contexts. Again, in Gram-negative bacteria, a pre-treatment using chloroform saturated buffer is needed to permeabilize the OM prior to start the kinetic assay [71]. Lysis is measured as a decrease of optical density over time which can be assessed by i) the steepest slope of the killing curve (i.e., unit is defined by ∆OD/min) [70,72] or by quantification of the amount of protein necessary to drop a percentage of the optical density relative to its initial value (i.e., unit is defined by a drop of density, e.g., of 50%) [10,54,73]. The use of different enzyme activity definitions makes comparison between studies difficult, therefore there is a need for a uniform unit definition for accurate quantification of muralytic activity [72]. It must be stressed that the turbidimetric assay cannot be applied to assess the killing activity of certain endolysins. A good example is the endolysin of *Paenibacillus larvae* phage phiIBB_Pl23 which can only reduce the optical density of cultures starting with an OD_620_ of 0.6 and not more [74]. Another example are endolysins of *Listeria* infecting phages that produce efficient lysis only if cells are previously frozen, probably to aid the degradation of their thick PG layers [60].

The changes in CFUs is often used to assess the enzymes’ antibacterial properties. The lytic agents are added to live bacterial suspensions of known concentrations, again generally prepared in buffers, and incubated during a period of time (usually minutes or hours). CFU counts are then compared with control groups and the antibacterial activity is usually expressed as a log reduction or a percentage of killing. Although this assay gives an important notion of the bacterial load that the enzyme is able to eliminate, it has the limitation of not being able to distinguish between not viable and viable but non-cultivable cells.

MIC assay aims at finding the lowest enzybiotic concentration that completely inhibits the visible planktonic growth of a microorganism. Variations of this assay include the minimum bactericidal concentration and the minimum biofilm eradication concentration, for determination of the minimum concentrations leading to undetectable CFUs after targeting suspended and sessile cultures, respectively [75,76]. In the MIC assay, serial dilutions of the lytic agent are distributed into wells of a microtiter plate and mixed with a defined concentration of cells prepared in culture media (usually up to 10^5^–10^6^ CFU/mL). MIC is given as the lowest enzyme concentration leading to no visible bacterial growth after overnight incubation. This data is useful to compare enzymes’ performance with antibiotics, which are routinely tested this way, providing also a starting point for setting doses to be used in in vivo assays [77]. MIC assays have been particularly used to assess the antimicrobial potential of engineered lytic enzymes and to study the synergistic effect when these are combined with conventional antibiotics [54,68,78].

Zymograms are a modified version of an SDS-PAGE gel where autoclaved bacterial cells or extracted PG are incorporated in the gel matrix to achieve haziness. After electrophoresis, the presence of PG-degrading enzymes is revealed by the appearance of transparent bands (lysis zones) after protein renaturation, which is achieved by SDS removal with rinsing water of buffer. This more hard-to-implement assay is more useful to pinpoint the enzymes according to their molecular weight and therefore also used to screen PG-degrading activity in blind expression libraries. Like the spot-on-lawn method however, the activity assessment is only qualitative [79,80].

### 3.2. In Vivo Performance Evaluation

The majority of enzybiotics is in pre-clinical phase trials and has been tested to target Gram-positive bacterial pathogens that include *Streptococcus* spp. [10,48,55,73,78,81,82,83,84,85,86,87,88,89,90,91,92,93], *S. aureus* [45,54,94,95,96,97,98,99,100,101,102,103,104,105,106,107], *Bacillus anthracis* [108,109] and *E. faecalis* [110] and more recently the Gram-negative pathogens *P. aeruginosa* [111] and *A. baumannii* [112,113]. According to literature, there is only one report showing the efficacy of a VAL (P128) against *S. aureus* in vivo. However, as discussed by São-José [51] there are several engineered chimeric enzymes sharing the ECD of Ply187, which is most probably a VAL of the staphylococcal phage 187 and not the phage’s endolysin as previously reported. Ply187-derived chimeolysins tested in vivo include Ply187AN-KSH3b, ClyH and ClyF [94,101,114,115].

Animal models of systemic infection have been the most used to study the in vivo potential of enzybiotics, which are administrated by parenteral way to treat bacteremia. A compilation of these in vivo experiments is presented in Table S1. Here, animals are challenged with deadly doses of bacteria, treated with endolysins few hours post infection and readouts account mostly for the number of surviving mice after treatment and the development of neutralizing antibodies. There are no standardized methods to assess endolysins’ performance in vivo, animals are challenged with different infectious doses of bacteria and different administration routes and timings are employed, which render the comparison between results difficult. Cpl-1 is a good example of mixed outcome reports (Table S1). In one study, 100% Cpl-1-treated mice survived when Cpl-1 was added intravenously 1 h-postinfection [73]. In a second independent study, only about 30% Cpl-1-treated mice survived when the enzyme was added intraperitoneally 1 h-post infection [78]. Another study demonstrated that Cpl-1 failed when added by intraperitoneal injection [48]. Nevertheless, other enzymes have repeatedly shown success in treatment of systemic infections when administered intraperitoneally, such as PlyG, MV-L, CF-301, Ply30 and PlyF307 targeting *B. anthracis*, *S. aureus, S. pyogenes, S. suis* and *A. baumannii*, respectively (Table S1). Most importantly, the majority of in vivo efficacy studies treat animals a few hours after initiation of infection, usually 1 h after bacterial inoculation (Table S1), therefore only mimicking treatment of early bacteremia, a rare clinical occurrence. In these assays, the enzybiotics essentially provide a protective effect, rather than functioning as an effective therapy against well-established infections. The few studies that report different administration times show that treatment fails when enzymes are added latter. For example, mice with pneumococcal bacteremia treated 1 h post infection with the endolysin Cpl-1 showed 100% survival compared to the 20% survival of the non-treated group [73]. However, in advanced bacteremia (5 h and 10 h post infection), the same enzyme was able to extend the lifespan but not to increase mice survival rates, when compared to control groups. LysGH15 is another extensively studied endolysin to control invasive MRSA-infected mice. In two independent studies, it was shown that either i.p. or i.v. single doses of LysGH15 (50 µg/mouse) were sufficient to protect mice against lethal systemic MRSA infections [103,116]. Nevertheless, it was also clear that survival rates were time dependent. LysGH15 could only rescue 100% of bacteremic mice if added 1 h after bacterial challenge, being the survival rate significantly decreased to 20% when added 4 h later. Other studies reporting enzyme administrations 1 h and 3 h-post infection have also shown a mild activity (Table S1). This is the case of plyF307 for instance, that when applied 1 h after infecting mice with *A. baumannii* resulted in 50% of survival compared with the 10% of survival of the control group [112]. The fact that endolysins when applied systemically could face short half-life, immunogenicity, allergenicity, proinflammatory responses and inability to target intracellular bacteria is well discussed elsewhere [50]. Herein we will focus on the lower efficiency of endolysins that is frequently observed under conditions promoting robust bacterial growth and which could explain some of their limitations observed in vivo.

## 4. In Vitro to in Vivo Translation

### 4.1. Hurdles

There are hundreds of reports supporting the antibacterial activity of endolysins in vitro and a considerable number of these enzymes have also been tested in vivo. Still, a major question remains: is the capacity to kill bacteria from without an intrinsic feature of endolysins and are they all suitable for use as enzybiotics? The use of endolysins as enzybiotics builds on the idea that they should be able to efficiently lyse bacteria from without, as long as contact to the CW is granted. However, this premise conflicts with the natural mode of action of endolysins, which have evolved to act from within and after holin-mediated collapse of the membrane potential, that is, after cell death (see Section 2.1). As such, the conditions used to evaluate the antibacterial efficacy of a given enzyme in vitro may have great impact on results and therefore caution should be taken when making extrapolations to in vivo scenarios.

Most in vitro studies to assess the bactericidal potential of enzybiotics employ target cells resuspended in buffered solutions, that is, in conditions not supporting bacterial growth. The few examples of enzymes tested in more complex and realistic bio-matrixes have shown limited or absence of activity (see below). Therefore, the enzybiotic antibacterial efficacy obtained in vitro may not be translated in the complex environments also observed in vivo. As already mentioned, the cases where in vitro efficiency seems to translate to in vivo are mostly observed when lytic enzymes are administrated to animals soon after bacterial challenge. Possibly, because bacteria are usually washed with buffers prior injecting them into the animals, cells may become in a transient “lag” state that is more susceptible to the enzymes’ lytic action (analogous to the in vitro lysis of buffer-suspended cells). In time, the bacterial cells eventually adapt to the new nutrient conditions in the host, becoming metabolic more active and eventually more resistant to the action of enzymes (which are known to fail when added several hours after bacterial challenge in systemic infections). Endolysins are also routinely characterized in vitro using exponential growth phase cells and not cells in older physiological states. In some cases, stationary-phase cells were shown to be less susceptible to the enzymes’ lytic action, probably due to cell envelope structural or chemical modification of the PG [65,92]. It is known that PG maturation and remodeling occurs in stationary cells and can involve the acetylation and/or *N*-deacetylation of glycan chains and amidation of peptides, modifications that may confer resistance to lytic enzymes [117]. The relevance of these observations when passing to in vivo scenarios is still unknown. These hurdles have been shown both with Gram-positive and Gram-negative pathogens.

Recent studies have highlighted the influence of the cell energy state and growth conditions in bacterial susceptibility to some endolysins. Lys170, an enterococcal endolysin harboring an N-terminal ECD and a C-terminal CBD, could efficiently lyse viable *E. faecalis* cells grown to exponential stage and suspended in a physiologic buffer before enzyme treatment [43]. However, the same Lys170 amounts failed to lyse or prevent growth of cells suspended in nutritional medium like TSB. Possible inhibition effects of TSB components were discarded by simultaneously treating cell suspensions with Lys170 and the lantibiotic nisin, which induces membrane pore formation and consequently cell death. Cells killed by the nisin action revealed to be fully susceptible to the Lys170 lytic action, indicating that TSB components did not significantly interfere with the endolysin activity. Authors hypothesized that it could be a result of the inability of the endolysin to act on actively growing cells, as buffers do not support cell growth.

The fact that dividing and fully energized bacteria are less susceptible to endolysin attack makes biological sense because, as already mentioned, these enzymes are naturally designed to cleave the PG of cells killed by the action of cognate holins. This hypothesis gained further support after studies with the endolysins of two well-known phages, the *Bacillus subtilis* phage SPP1 and the *S. aureus* phage phi11 [118]. The investigation showed that conditions inducing cytoplasmic membrane depolarization (holin action, membrane ionophores or nutrient depletion) could dramatically increase bacterial susceptibility to the endolysins attacking the CW either from within or from without. The results of this work also indicated that the capacity of energized cells to counteract endolysin attack may vary significantly depending on the tested bacterium/endolysin pair and growth conditions. Overall, the idea that emerged is that the lytic efficacy of endolysins may be significantly conditioned by the energetic state of target cells, something that should be verified when exploring these agents as enzybiotics. As discussed in the referred studies, a relationship between the cytoplasmic membrane energy state and the susceptibility of bacteria to PG-degrading enzymes (either endogenous or exogenous) has been reported in many different contexts.

Although the OM of Gram-negative bacteria is generally viewed as a barrier that blocks access to the CW of exogenously-added enzymes, several examples of endolysins that spontaneously cross the OM and exert lytic activity were reported [21,62,63,119,120]. Similarly, these enzymes have also been tested in artificial conditions using bacteria suspended in buffer and activity is not reproduced when tested on different environments. For instance, we have recently cloned and tested the antibacterial activity of the *Acinetobacter* phage Vb_MAb_B9 endolysin in different biomatrixes. The endolysin was able to reduce more than three orders of magnitude the cell counts of a bacterial suspension prepared in Hepes. However, in other media like PBS or TSB it lacked antibacterial activity [121] A peptide-modified endolysin (PlyA) was shown to reduce more than five orders of magnitude the counts of logarithmic phase *A. baumannii* cells suspended in Tris-HCl buffer. Yet, it was completely inactive when tested in biomatrixes such as pasteurized milk, human serum and different bacterial culture media [122]. In addition, PlyA could kill buffer-suspended, *A. baumannii* stationary cells only when in presence of OM permeabilizers. Another example is the *A. baumannii* phage endolysin PlyF307, which was able to reduce more than 3 orders of magnitude the cells collected from exponentially growing cultures but only one order of magnitude in case of stationary phase bacteria [112]. Therefore, several reports indicate that endolysins’ high performance in vitro might not translate into in vivo efficacy, where cells are not in the optimal conditions for enzybiotic activity.

### 4.2. Possible Solutions

#### 4.2.1. Complementary Screening Tests to Search for the Best Enzybiotic

To better assess the enzybiotics’ potential for in vivo applications, their performance should be evaluated not only under different ranges of pH, temperatures and ionic strengths but also using different environment conditions and cells in different physiological states mimicking a more likely in vivo scenario. Furthermore, experiments using human serum and simpler animal models (*C. elegans*, *G. mellonella*, *Drosophila melanogaster* or zebrafish) [123,124,125,126] could also be considered as an alternative to evaluating the antimicrobial activity of enzymes considered to treat systemic infections, before using more complex mammalian systems, such as murine models.

#### 4.2.2. Using VALs

VALs, as opposite to endolysins, need to maintain activity in several environmental conditions and against different physiological states of the host bacterium to allow phage infection and proliferation. With this hypothesis in mind, Proença et al. [43] generated a chimeric protein (EC300) harboring the M23 ECD of the VAL Orf73 and the CBD of the endolysin Lys170, both from the *E. faecalis* phage F170/08. In contrast to the endolysin, the chimeolysin showed efficient lytic action from outside against dense cell cultures in nutritional medium. Another important study in this field was the characterization of the coliphage T7 VAL. Despite the predicted role of this enzyme to help the phage tail to penetrate the PG-mesh structure at onset of infection, its function is dispensable when phage infects the host under optimal laboratory conditions [14]. Interestingly, the T7 VAL function is only required when host cells have the PG highly cross-linked, namely when in stationary or when growing at low temperatures. This demonstrates that VALs are adapted to cleave the PG of host cells under physiological states different from logarithmic growth, which is routinely tested but not always present in human infections. This hypothesis is further supported by the characterization of the lactococcal phages Tuc2009 and TP901-1 VALs, both harboring a M23 peptidase ECD and which were proved to be essential for phages to efficiently infect stationary phase cells [30]. As already noted, the M23 ECD is almost absent in endolysins but seems to be frequently present on VALs and on other non-viral PG-degrading enzymes naturally designed to act on the bacterial CW from the outside (bacteriolysins like lysostaphin and enterolysin-A). This probably indicates that this ECD and other domains present in VALs might have evolved to efficiently compromise the PG of bacterial cells from without to maintain phage infectivity. If true, VALs may possess superior antibacterial activity than some endolysins and play a more decisive role for applications in vivo against pathogenic bacteria. Moreover, ECDs from VALs may present increased thermostability when compared to those derived from endolysins [32,37,42,71], which may result in prolonged shelf life.

#### 4.2.3. Engineered Lytic Enzymes

The possibility of modifying native VALs and endolysins, or of using them as scaffolds to create completely new enzybiotics, may provide additional solutions to overcome the described obstacles of the field. Protein engineering is a growing trend in the area of phage lytic enzymes as it has been showing the potential to generate enzymes with several improved features. These include not only enhanced bactericidal activity against bacteria in different metabolic states and environments but also expansion of the lytic spectrum and increased solubility, stability and circulating half-life. As already mentioned, there are several strategies to tailor enzymes, namely domain deletion, addition, swapping and site-directed modifications. Aleatory exchange of functional domains or random mutagenesis, coupled to high-throughput screening methods, have also been used to select enzybiotics with upgraded characteristics (for a recent review on enzybiotics engineering see [51]). These approaches could benefit from ex-vivo screening procedures closely mimicking the targeted human conditions. The VAL-derived chimeolysins EC300 and P128 are good examples of novel enzybiotics that efficiently kill cells actively growing in complex media [43]. This breakthrough encourages the creation of new tailor-made proteins using not only VAL domains but also other agents known to act from without such as bacteriocins, antimicrobial peptides and bacteriolysins. Recent developments have also uncovered the potential of engineered, phage-derived lytic enzymes to target intracellular pathogens [95,127]. In summary, artificial enzybiotics may provide a way to circumvent some of the limitations described for native enzymes when applied in vivo.

## 5. Conclusions and Perspectives

The demonstration of the bactericidal activity of phage PG-degrading enzymes has sparked the interest for their exploration as alternatives or complements to existing antibiotics, particularly in the current context of uncontrolled dissemination of antimicrobial drug resistance. These enzybiotics are nontoxic, they usually have a narrow lytic spectrum and display quick bactericidal kinetics with low probability of resistance development. The first demonstration of the therapeutic potential of phage endolysins was followed by almost two decades dedicated to the discovery of new lytic agents and their study in vitro and in animal models. Therefore, it was expected that a higher number of these enzymes would have already made the transition from the proof-of-principle phase to the clinical stage. After carefully reviewing the literature, we came to the conclusion that most endolysins have been tested in conditions that are a poor approximation of real life bacterial infection scenarios. This might account in part for some failures when the enzymes are tested in vivo and various suggestions are given for testing them in more complex and realistic environments. In addition, we suggest VALs as another class of PG-degrading enzymes that deserve further attention as they seem to present natural advantages when compared to endolysins. Finally, in recent years this field of research has been gradually moving to the modification of native lytic enzymes and to the generation of new and improved enzybiotics through protein engineering techniques. This new era has been delivering promising results and clues to overcome the limitations of native enzymes.

## Supplementary Materials

The following are available online at http://www.mdpi.com/1999-4915/10/6/292/s1. Table S1: Compilation of phage lytic enzymes that have been tested in animal models of human infections.
