# Supplementary material for: Phage-Derived Peptidoglycan Degrading Enzymes: Challenges and Future Prospects for In Vivo Therapy
Source: Viruses. 2018 May 29;10(6):292. doi: 10.3390/v10060292 (PMC6024856; doi:10.3390/v10060292)
Supplement: Supplementary file 1 [file viruses-10-00292-s001.docx]

**Table S1. Compilation of phage lytic enzymes that have been tested in animal models of human systemic infections.**

| **Enzybiotic** | **Native / Engineered** | **Bacterial pathogen** | **Animal**  **Model^1^** | **Administration**  **route1^2^** | **Regimen**  **(Dose and time of treatment)** | **Main results** | **Refs** |
| --- | --- | --- | --- | --- | --- | --- | --- |
| Cpl-1 | N | *S. pneumoniae* | Bacteraemia | i.p. | Single dose (0.4 or 1 mg/kg of mouse)  1 h-postinfection | 0% buffer-treated and 0% enzybiotic-treated mice survived.  Combination with daptamyxin resulted in ≥ 80% survival. | [90] |
|  | N | *S. pneumoniae* | Bacteraemia | i.v. | Single or multiple dose (2 mg/mouse)  1 h-postinfection or 5 and 10-postinfection | 20% buffer-treated and 100% enzybiotic-treated mice survived.  In advance bacteraemia (5 and 10 h after infection) all mice died. | [73] |
| Cpl-1, Pal | N | *S. pneumoniae* | Sepsis | i.p. | Single dose (5 to 200 µg/mouse)  1 h-postinfection | 0% buffer-treated and 100% C-treated mice survived with 200 µg of either enzyme. Survival rate was 0% with 5 µg. Cpl1 synergizes with Pal. | [49] |
| Cpl-771, Cpl-1 | E, N | *S. pneumoniae* | Bacteraemia | i.p. | Single dose (25 to 500 µg/mouse)  1 h-postinfection | 0% buffer-treated, ≥45% cpl-771-treated and ≥20% Cpl-1-treated mice survived. With highest doses, 100% and 30% survived, respectively.  Cpl-771 was 50% superior than Cpl-1. | [79] |
| Ply30 | N | *S. suis / S. equi* | Bacteraemia | i.p. | Single dose (2 mg/mouse)  1 h-postinfection | 0% buffer-treated and ≥80% enzybiotic-treated mice survived within 96 h post treatment. | [91] |
| PlyPy | N | *S. pyogenes* | Bacteraemia | i.p. | Single dose (0.25 or 0.5 mg/mouse)  3 h-postinfection | 17% buffer-treated and ≥90% enzybiotic-treated mice survived 72 h post treatment. | [92] |
| PlySK1249 | N | *S. agalactiae* | Bacteraemia | i.p. | Single or multiple dose (22.5 to 45 mg/kg of mouse)  1 h or 2, 20 and 24 h-postinfection | No differences between buffer and treated group when administrated 1 h-postinfecton. Only consecutive doses (2, 20 and 24 h after challenge), treatment recused 60% more mice then control group. | [93] |
| ClyR | E | *S. agalactiae* | Bacteraemia | i.p. | Single dose (25 to 40 mg/kg of mouse)  3 h-postinfection | 0% non-treated and ≥25% enzybiotic-treated mice survived. Total protection was obtained with the highest ClyR dose. | [94] |
| ClyS | E | *S. aureus* | Septicaemia | i.p. | Single dose (2 mg/mouse)  3 h-postinfection | 0% buffer-treated and 88% enzybiotic-treated mice survived.  CF-301 has a synergistic effect with oxacillin. | [55] |
| ClyH | E | *S. aureus* | Bacteraemia | i.p. | Single dose (450 or 900 µg/mouse)  3 h-postinfection | 0% buffer-treated and ≥66.7% enzybiotic-treated mice survived. A 100% survival was reach with highest dose. Daily injections of ClyH did not cause harmful effects. | [102] |
| MR-10 | N | *S. aureus* | Bacteraemia | subcutaneously | Single dose (50 µg/mouse)  3 h-postinfection | 0% buffer-treated and 100% enzybiotic and -treated mice survived.  Individual therapy (MR-10 or minocycline) resulted in 35% survival. | [103] |
| LysGH15 | N | *S. aureus* | Bacteraemia | i.p. | Single dose (5 to 100 µg/mouse)  1, 2, 3 or 4 h-postinfection | 0% non-treated and 100% enzybiotic-treated mice survived with ≥50 µg.  Prolonged administrations (2, 3 and 4 h) had 40% to 0% survival rate. | [104] |
| CF-301 | N | *S. aureus* | Bacteraemia | i.p. | Single dose (0.25 to 5 mg/kg of mouse)  3 h-postinfection | 0% buffer-treated and ≥20% enzybiotic-treated mice survived for ≥2.5 mg/kg doses. A maximum 70% survival was reached with the highest dose.  CF-301 synergizes with vancomycin or with daptomycin. | [105] |
|  |  | *S. aureus* and  *S. pyogenes* | Bacteraemia | i.p. | Single dose (2 to 4 mg/kg of mouse)  3 h-postinfection | 0% buffer-treated and 92% enzybiotic-treated mice survived from mixed infection. PlyC or ClyS added to in the same concentrations, failed. | [106] |
| MV-L | N | *S. aureus* | Bacteraemia | i.p. | Single dose (500 U/mouse)  30 or 60 min-postinfection | 0% buffer-treated and ≥60% enzybiotic-treated mice survived when added 60 min after challenge. Fully protection was reached at 30 min. | [46] |
| SAL-1 | N | *S. aureus* | Bacteraemia | i.v. | Multiple dose (12.5 to 25 mg/kg of mice)  1, 25 and 49 h-postinfection | 20% non-treated and ≥93.3% enzybiotic-treated mice survived. Bacterial counts were significantly reduced in the bloodstream and splenic tissue. | [107] |
| 8 enzybiotics  and lysostaphin | N, E | *S. aureus* | Bacteraemia | i.p. | Single dose (200 µg/mouse)  30 min-postinfection | 30% buffer-treated and 100% enzybiotic-treated mice survived.  Twort, phiSH2 and P68 had lower survival rates of 50%, 60% and 20%. | [108] |
| PlyG | N | *B. anthracis* | Sepsis | i.p. | Single dose (50 U/mouse)  15 min-postinfection | 0% buffer-treated and 68.4% enzybiotic-treated mice survived.  No toxicity was detected. | [110] |
| LysEF-P10 | N | *E. faecalis* | Bacteraemia | i.p. | Single dose (1 to 10 µg/mouse)  1 h-postinfection | 0% buffer-treated and 100% enzybiotic-treated mice survived for ≥ 5µg doses. Lowest dose (1 µg) only rescued 20% of mice.  LysEF-P10 triggers antibodies but does not abolish the enzymatic activity. | [111] |
| PlyF307 | N | *A. baumannii* | Sepsis | i.p. | Single dose (1 mg/mouse)  2 h-postinfection | 10% buffer-treated and 50% enzybiotic-treated mice survived.  Enzybiotic also removed biofilms in a *in vivo* catheter model. | [113] |

^1^  murine model unless stated otherwise;

^2^ i.v., intravenously; i.p., intraperitoneal;
